# The optimal extent of gastrectomy for middle-third gastric cancer: distal subtotal gastrectomy is superior to total gastrectomy in short-term effect without sacrificing long-term survival

**DOI:** 10.1186/s12885-017-3343-0

**Published:** 2017-05-19

**Authors:** Xin Ji, Yan Yan, Zhao-De Bu, Zi-Yu Li, Ai-Wen Wu, Lian-Hai Zhang, Xiao-Jiang Wu, Xiang-Long Zong, Shuang-Xi Li, Fei Shan, Zi-Yu Jia, Jia-Fu Ji

**Affiliations:** 1 Key laboratory of Carcinogenesis and Translational Research (Ministry of Education), Department of Gastrointestinal Surgery, Peking University Cancer Hospital & Institute, No. 52 Fucheng Road, Haidian District, Beijing, 100142 China; 2 Key laboratory of Carcinogenesis and Translational Research (Ministry of Education), Department of Endoscopy, Peking University Cancer Hospital & Institute, Beijing, China

**Keywords:** Middle-third gastric cancer, Distal subtotal gastrectomy, Total gastrectomy, Overall survival

## Abstract

**Background:**

The optimal extent of gastrectomy for middle-third gastric cancer remains controversial. In our study, the short-term effects and longer-term survival outcomes of distal subtotal gastrectomy and total gastrectomy are analysed to determine the optimal extent of gastrectomy for middle-third gastric cancer.

**Methods:**

We retrospectively collect and analyse clinicopathologic data and follow-up outcomes from a prospectively collected database at the Peking University Cancer Hospital. Patients with middle-third gastric adenocarcinoma who underwent curative resection are enrolled in our study.

**Results:**

We collect data of 339 patients between January 2005 and October 2011. A total of 144 patients underwent distal subtotal gastrectomy, and 195 patients underwent total gastrectomy. Patients in the total gastrectomy group have longer operative duration (*P* < 0.001) and postoperative hospital stay (*P* = 0.001) than those in the distal subtotal gastrectomy group. In the total gastrectomy group, more lymph nodes are harvested (*P* < 0.001). Meanwhile, the rate of postoperative complications is lower in the distal subtotal gastrectomy group than in the total gastrectomy group (8% vs 15%, *P* = 0.047). Further analysis demonstrates that the rate of anastomosis leakage is lower in the distal subtotal gastrectomy group than in the total gastrectomy group (0% vs 4%, *P* = 0.023). Kaplan–Meier (log rank test) analysis shows a significant difference in overall survival between the two groups. The 5-year overall survival rates in the distal subtotal gastrectomy and total gastrectomy groups are 65% and 47%, respectively (*P* < 0.001). Further stage-stratified analysis reveals that no statistical significance exists in 5-year survival rate between the distal subtotal gastrectomy and total gastrectomy groups at the same stage. Multivariate analysis shows that age (*P* = 0.046), operation duration (*P* < 0.001), complications (*P* = 0.037), usage of neoadjuvant chemotherapy (*P* < 0.001), tumor size (*P* = 0.012), presence of lymphovascular invasion (*P* = 0.043) and N stage (*P* < 0.001) are independent prognostic factors for survival.

**Conclusions:**

For patients with middle-third gastric cancer, distal subtotal gastrectomy shortens the operation duration and postoperative hospital stay and reduces postoperative complications. Meanwhile, the long-term survival of patients with distal subtotal gastrectomy is similar to that of those with total gastrectomy at the same stage. The extent of gastrectomy for middle-third gastric cancer is not an independent prognostic factor for survival.

## Background

Gastric cancer is a severe problem worldwide. It is the fourth most common cancer and the second leading cause of cancer death globally. According to recent reports, nearly 950,000 new cases are diagnosed each year, and 720,000 patients with gastric cancer died from gastric cancer in 2012 [1, 2]. Although gastric cancer is not a common cancer in North America or most Western European areas, the burden of gastric cancer is still very high in Eastern Asia, Central and Eastern Europe, and Latin America [3]. Specifically, more than 50% of patients arise in the Eastern Asian area.

Surgery is the mainstay in the multidisciplinary treatment for gastric cancer. Adequate surgical resection is the only potentially curative method for gastric cancer [4, 5]. Surgery for gastric cancer must ensure the complete removal of the tumor and potentially metastatic lymph nodes. Meanwhile, the intraoperative and postoperative safety and postoperative quality of life should be under consideration before surgeons determine the surgical treatment strategy. The extent of surgical resection is determined by tumor stage, location, size, histological type and some other clinicopathological characteristics. An adequate gastrectomy is defined as complete resection of the primary tumor with negative resection margins. According to the latest Japanese gastric cancer treatment guidelines published in 2016, the standard surgical procedure for tumor with clinically positive lymph nodes or tumor invading to or deeper than the muscularis propria is either distal subtotal gastrectomy or total gastrectomy [6, 7]. Distal subtotal gastrectomy could be selected when a satisfactory proximal resection margin can be achieved. As a result, for tumors located in the upper third of the stomach, proximal subtotal gastrectomy or total gastrectomy is recommended, depending on the depth of tumor invasion [8]. With regard to lower-third gastric cancer, distal subtotal gastrectomy is the optimal surgical procedure suggested by previous studies [9, 10]. Nonetheless, the extent of gastrectomy for middle-third gastric cancer remains controversial. Some studies have recommended total gastrectomy as the standard procedure because of its potential for improved long-term survival [11, 12]. Considering the better intraoperative and postoperative safety and quality of life, distal subtotal gastrectomy has been reported to be an alternatively curative treatment for middle-third gastric cancer [13].

The short-term effect and long-term prognosis of different extents of gastrectomy for middle-third gastric cancer have not been well evaluated until now. In our study, we therefore analyse the intraoperative and postoperative effects and long-term survival outcomes of patients with middle-third gastric cancer who underwent different extents of gastrectomy.

## Methods

### Patients

This study is carried out under the approval of the Ethics Committee of Peking University Cancer Hospital. Each patient within this study signed informed consent. We retrospectively collect clinicopathological data from a prospectively collected database at the Peking University Cancer Hospital. Between January 2005 and October 2011, a total of 339 patients with middle-third gastric adenocarcinoma who underwent curative resection are enrolled in our study. We adopt the Japanese definition of three regions of the stomach in this study. The stomach is anatomically divided into three portions, the upper, middle, and lower parts, by lines connecting the trisected points on the lesser and greater curvatures. Tumors are described by the parts involved. If more than one part is involved, all involved portions are recorded in descending order of the degree of involvement, with the part containing the bulk of the tumor first [14]. In our study, the centre of the primary tumor in all patients is located in the middle third of the stomach, and the tumors do not invade beyond the border between the upper and middle third of the stomach. In other words, all tumors are located in the middle third or middle-lower third of the stomach according to the Japanese classification of gastric carcinoma [14]. The initial diagnosis was confirmed by endoscopic biopsy examination. Clinical staging was evaluated with ultrasound endoscopy of the stomach, abdominal and pelvic computed tomography scans, and laparoscopic exploration. The stage was classified based on the 7th edition Union for International Cancer Control (UICC)/American Joint Committee on Cancer (AJCC) TNM staging system [7]. Patients with other types of gastric carcinoma, such as gastrointestinal stromal tumors or lymphoma, are excluded from this study.

### Surgical treatments

All of the patients underwent laparoscopic exploration to exclude distant metastatic disease. After that, surgeons performed distal subtotal gastrectomy or total gastrectomy. The principle of surgery was mainly based on the Japanese gastric cancer treatment guidelines [8, 15]. Distal subtotal gastrectomy was a choice if a negative proximal resection margin could be obtained. The following rules of resection margin were adopted during the operation. The proximal resection margin was at least 3 cm for tumors invading to or deeper than the muscularis propria with an expansive growth pattern, or at least 5 cm for those with an infiltrative growth pattern. For tumors limited to mucosa or submucosa, a gross resection margin of 2 cm was obtained. If the above-mentioned criteria could not be fulfilled, frozen section examination of the proximal resection margin was completed to secure a negative resection margin. For cT1N0 tumors, D1 or D1+ lymph node dissection was conducted. For lymph node-positive or T2-T4 tumors, standard D2 lymph node dissection was performed.

Postoperative recovery was conducted by medical care professionals. Before the patient could leave the hospital, the discharge criteria had to be fulfilled. These criteria include the following: absence of subjective complaints, tolerance of solid oral intake, return of bowel function, absence of intravenous fluids/medications, adequate mobility of daily living and self-care (e.g., go to the toilet, dress, shower, etc.), adequate pain control on oral analgesia only, adequate wound condition, removal of the drainage tube, absence of infectious complications, absence of postoperative complications, absence of abnormal physical signs or laboratory tests (e.g., pulse, body temperature, white blood cell count, serum haemoglobin, etc.), acceptance of discharge, and an adequate home/social condition. In our study, adjuvant chemotherapy was carried out in patients who were identified as pathological T3/4 or metastasis in lymph nodes. Adjuvant chemotherapy was usually performed with cisplatin-based or 5-fluorouracil-based systemic therapy. However, radiotherapy was not used for all patients in our study.

### Clinicopathologic parameters and follow-up

The clinicopathological data collected from the database include the extent of gastrectomy, age, sex, body mass index (BMI), usage of neoadjuvant and adjuvant chemotherapy, degree of differentiation, presence of lymphovascular invasion, tumor size, tumor location, multi-tumor presence, depth of tumor invasion, number of harvested and metastatic lymph nodes, length of proximal resection margin, postoperative complications, reoperation, mortality, length of postoperative hospital stay, operation duration, blood loss volume, and survival outcome. The terminology used in this study is based on the Japanese classification of gastric carcinoma [14]. Follow-up was carried out mainly by means of telephone interviews, E-mail communication, or outpatient reviews. The last follow-up was conducted on October 27, 2016.

### Statistical analysis

All statistical analyses are performed using IBM SPSS Statistics 20.0 software (SPSS Inc., Armonk, NY). For quantitative variables, a normal distribution is verified. Variables with a normal distribution are expressed as the mean ± standard deviation and tested by a *t* test between groups. If not, the variables are expressed as medians with 25–75% ranges and tested by a Kruskal–Wallis non-parametric test. For categorical data, the chi-squared test or Fisher’s exact test is performed. Kaplan–Meier estimation and log-rank tests are performed to compare survival. A Cox proportional hazards regression model is used to verify independent prognostic factors by univariate and multivariate analysis. *P* < 0.05 (two-sided) is considered significant in the statistical analysis.

## Results

### Clinicopathologic parameters

A total of 339 patients are enrolled in this retrospective study, and all of these patients are divided into a distal subtotal gastrectomy group (*n* = 144) or a total gastrectomy group (*n* = 195). The clinicopathological parameters are compared between the two groups. Age, sex, BMI, degree of differentiation and multi-tumor presence are comparable between the groups. More patients in the total gastrectomy group receive neoadjuvant chemotherapy (*P* < 0.001). More patients in the total gastrectomy group have lymphovascular invasion (*P* = 0.015). Moreover, more patients in the total gastrectomy group are at a later T stage (*P* < 0.001), N stage (*P* = 0.027), and have larger tumor size (*P* < 0.001). More patients in the total gastrectomy group receive adjuvant chemotherapy (*P* < 0.001). More patients in the distal subtotal gastrectomy group have tumors invading into the lower third of the stomach (*P* = 0.038; Table 1). From these results, it seems that surgeons are inclined to choose total gastrectomy if the tumor is diagnosed as a relatively later-stage disease.

**Table 1:** Patients’ clinicopathological parameters

Clinicopathological parameters	Distal subtotal gastrectomy (*n* = 144), *n* (%)	Total gastrectomy (*n* = 195), *n* (%)	*P* value
Gender			0.641
Male	94 (65)	132 (68)	
Female	50 (35)	63 (32)	
Age			0.354
≤ 60	87 (60)	108 (55)	
> 60	57 (40)	87 (45)	
Body mass index			0.797
< 19	19 (14)	27 (14)	
~ <25	84 (60)	111 (58)	
~ <30	31 (22)	48 (25)	
≥ 30	5 (4)	4 (2)	
Neoadjuvant chemotherapy			<0.001
No	117 (81)	123 (63)	
Yes	27 (19)	72 (37)	
Differentiation			0.117
Well	16 (11)	21 (11)	
Moderate	63 (44)	67 (34)	
Poor	65 (45)	107 (55)	
Lymphovascular invasion			0.015
No	89 (63)	96 (50)	
Yes	52 (37)	97 (50)	
Tumor size			<0.001
≤ 5 cm	109 (78)	92 (47)	
> 5 cm	31 (22)	102 (53)	
Location			0.038
Middle	88 (61)	140 (72)	
Middle-lower	56 (39)	55 (28)	
Multi-tumor			0.699
No	139 (98)	192 (99)	
Yes	3 (2)	3 (1)	
Adjuvant chemotherapy			<0.001
Yes	107 (74)	186 (95)	
No	37 (26)	9 (5)	
T stage			<0.001
T1	37 (26)	12 (6)	
T2	19 (14)	11 (6)	
T3	3 (2)	10 (5)	
T4	82 (58)	182 (83)	
N stage			0.027
N0	50 (39)	52 (27)	
N1	26 (20)	30 (16)	
N2	19 (15)	32 (17)	
N3	33 (26)	77 (40)	

### Intraoperative and postoperative parameters

Intraoperative and postoperative parameters are compared between the two groups (Table 2). The results show that the length of proximal resection margin, blood loss volume, rate of reoperation and postoperative mortality have no significant differences between the two groups. In the total gastrectomy group, more lymph nodes are harvested. The median numbers of dissected lymph nodes in the distal subtotal gastrectomy and total gastrectomy groups are 26 and 31, respectively (*P* < 0.001). Patients in the total gastrectomy group have a longer operative duration than those in the distal subtotal gastrectomy group. The median operation durations in the total gastrectomy and distal subtotal gastrectomy groups are 240 min and 180 min, respectively (*P* < 0.001). Patients in the distal subtotal gastrectomy group have a shorter postoperative hospital stay. The median postoperative hospital stays in the distal subtotal gastrectomy and total gastrectomy groups are 12 days and 14 days, respectively (*P* = 0.001). Meanwhile, the rate of postoperative complications is lower in the distal subtotal gastrectomy group than in the total gastrectomy group (8% vs 15%, *P* = 0.047). Further analysis demonstrates that the rate of anastomosis leakage is lower in the distal subtotal gastrectomy group than in the total gastrectomy group (0% vs 4%, *P* = 0.023). The rates of bleeding, obstruction, peritoneal abscess, abdominal infection, pancreatic fistula and anastomosis stricture are all comparable between the groups (Table 2).

**Table 2:** Patients’ intraoperative and postoperative parameters

Intraoperative and postoperative parameters	Distal subtotal gastrectomy	Total gastrectomy	*P* value
Proximal resection margin, cm, median (25–75% range)	5.0 (5.0–5.0)	5.0 (4.0–6.0)	0.939
Total number of dissected lymph nodes, median (25–75% range)	26 (18–34)	31 (23–43)	<0.001
Operation duration, min, median (25–75% range)	180 (154–213)	240 (190–270)	<0.001
Blood loss volume, ml, median (25–75% range)	150 (100–200)	150 (100–200)	0.178
Postoperative hospital stay, days, median (25–75% range)	12.0 (10–15.3)	14.0 (11.0–19.5)	0.001
Complication rate, *n* (%)	12 (8)	29 (15)	0.047
Bleeding, *n* (%)	2 (1)	7 (4)	0.311
Anastomosis leakage, *n* (%)	0 (0)	8 (4)	0.023
Obstruction, *n* (%)	1 (1)	1 (1)	1.000
Peritoneal abscess, *n* (%)	5 (4)	13 (7)	0.195
Abdominal infection, *n* (%)	6 (4)	17 (9)	0.100
Pancreatic fistula, *n* (%)	0 (0)	4 (2)	0.140
Anastomosis stricture, *n* (%)	0 (0)	1 (1)	1.000
Reoperation, *n* (%)	3 (2)	7 (4)	0.527
Mortality rate, *n* (%)	0 (0)	3 (2)	0.264

### Survival results

**Fig. 1:**
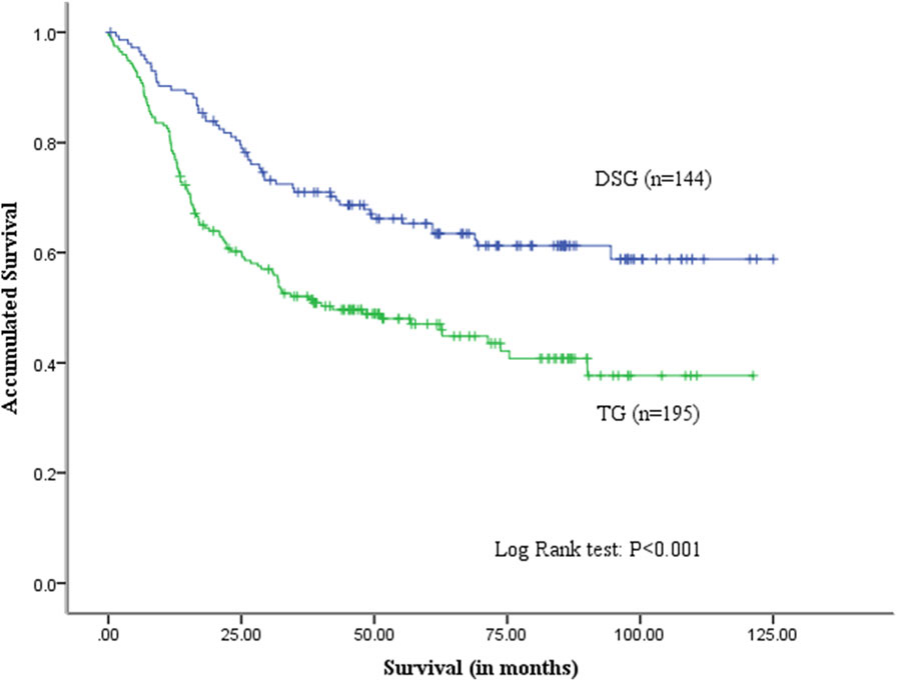
Overall survival curves of patients in the distal subtotal gastrectomy and total gastrectomy groups. Overall survival is better in the distal subtotal gastrectomy group than the total gastrectomy group. The 5-year survival rates in the distal subtotal gastrectomy and total gastrectomy groups are 65% and 47%, respectively (*P* < 0.001)

**Fig. 2:**
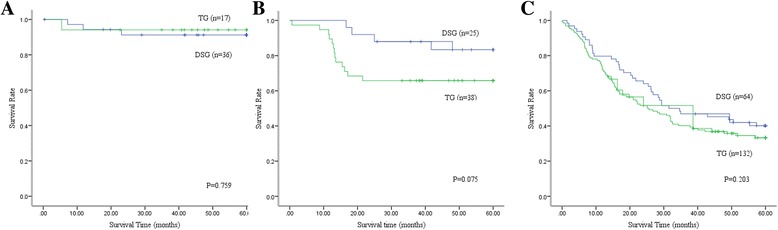
Stage-stratified survival curves of patients in the distal subtotal gastrectomy and total gastrectomy groups. In stage-stratified subgroup analysis of stage I, the 5-year survival rates in the distal subtotal gastrectomy and total gastrectomy groups are 91% and 94%, respectively (*P* = 0.759; (**a**). For patients at stage II, the 5-year survival rates in the distal subtotal gastrectomy and total gastrectomy groups are 83% and 66%, respectively (*P* = 0.075; **(b**). In the subgroup analysis of stage III, the 5-year survival rates in the distal subtotal gastrectomy and total gastrectomy groups are 40% and 33%, respectively (*P* = 0.203; (**c**)

The median follow-up time is 41.8 months (range: 1–125 months). The overall survival is better in the distal subtotal gastrectomy group than in the total gastrectomy group (*P* < 0.001; Fig. 1). The 5-year overall survival rates in the distal subtotal gastrectomy and total gastrectomy groups are 65% and 47%, respectively (*P* < 0.001). In consideration of the clinicopathological differences existing between the groups, stage-stratified subgroup analysis is carried out. The 5-year survival rate is compared between the groups at the same pathological stage. In the subgroup analysis of patients with stage I, the 5-year survival rates in the distal subtotal gastrectomy and total gastrectomy groups are 91% and 94%, respectively (*P* = 0.759; Fig. 2a). For patients with stage II gastric cancer, the 5-year survival rates in the distal subtotal gastrectomy and total gastrectomy groups are 83% and 66%, respectively (*P* = 0.075; Fig. 2b). In the subgroup analysis of patients with stage III gastric cancer, the 5-year survival rates in the distal subtotal gastrectomy and total gastrectomy groups are 40% and 33%, respectively (*P* = 0.203; Fig. 2c). The results demonstrate that the 5-year survival rates are comparable between the distal subtotal gastrectomy and total gastrectomy groups at the same stage.

All of the clinicopathological and perioperative parameters are included in the univariate analysis to identify the prognostic factors for survival. The results show that the extent of gastrectomy (*P* < 0.001), tumor location (*P* = 0.006), degree of differentiation (*P* = 0.019), blood loss volume (*P* = 0.005), age (*P* = 0.002), operation duration (*P* < 0.001), complications (*P* = 0.015), usage of neoadjuvant chemotherapy (*P* < 0.001), tumor size (*P* < 0.001), presence of lymphovascular invasion (*P* < 0.001), usage of adjuvant chemotherapy (*P* < 0.001), T stage (*P* < 0.001) and N stage (*P* < 0.001) are all prognostic factors for survival (Table 3). Since the usage of adjuvant chemotherapy is highly correlated with the pathological T stage and N stage, it is not included in the multivariate analysis. Subsequently, the above-mentioned factors are included in a multivariate analysis. The results show that age (*P* = 0.046), operation duration (*P* < 0.001), complications (*P* = 0.037), usage of neoadjuvant chemotherapy (*P* < 0.001), tumor size (*P* = 0.012), presence of lymphovascular invasion (*P* = 0.043) and N stage (*P* < 0.001) are all independent prognostic factors for survival (Table 4).

**Table 3:** Univariate analysis of prognostic factors for survival

Variable		Univariate, hazard ratio (95% Confidence interval)	*P* value
Gender	Male	1	0.531
	Female	0.898 (0.641, 1.258)	
Body mass index	<19	1	0.841
	~ < 25	0.990 (0.626, 1.565)	0.964
	~ < 30	1.095 (0.656,1.830)	0.728
	≥30	0.665 (0.200,2.217)	0.507
Postoperative hospital stay		1.001 (0.990, 1.012)	0.895
Reoperation	No	1	0.726
	Yes	1.172 (0.481, 2.859)	
Multi-tumor	No	1	0.147
	Yes	0.259 (0.036, 1.850)	
Total number of dissected lymph nodes		1.008 (0.999, 1.018)	0.086
Proximal resection margin		0.943 (0.851, 1.044)	0.257
Gastrectomy	Distal subtotal gastrectomy	1	<0.001
	Total gastrectomy	1.894 (1.359, 2.640)	
Location	Middle	1	0.006
	Middle-lower	1.565 (1.137, 2.153)	
Differentiation	Well	1	0.019
	Moderate	1.032 (0.580, 1.837)	0.914
	Poor	1.609 (0.932, 2.780)	0.088
	Blood loss volume	1.001 (1.000, 1.002)	0.005
Age	≤60	1	0.002
	>60	1.654 (1.210, 2.260)	
Operation duration		1.003 (1.001, 1.005)	<0.001
Complications	No	1	0.015
	Yes	1.708 (1.104, 2.642)	
Neoadjuvant chemotherapy	No	1	<0.001
	Yes	1.791 (1.297, 2.475)	
Adjuvant Chemotherapy	No	1	<0.001
	Yes	10.850 (3.459, 34.033)	
Tumor size	≤5 cm	1	<0.001
	>5 cm	2.828 (2.052, 3.897)	
Lymphovascular invasion	No	1	<0.001
	Yes	2.622 (1.902, 3.615)	
T stage	T1	1	<0.001
	T2	3.961 (1.024, 15.322)	0.046
	T3	9.944 (2.485, 39.790)	0.001
	T4	12.988 (4.136, 40.782)	<0.001
N stage	N0	1	<0.001
	N1	2.542 (1.425, 4.532)	0.002
	N2	3.045 (1.690, 5.487)	<0.001
	N3	6.562 (4.017, 10.719)	<0.001

**Table 4:** Multivariate analysis of prognostic factors for survival

Variable		hazard ratio (95% Confidence interval)	*P* value
Gastrectomy	Distal subtotal gastrectomy	1	0.942
	Total gastrectomy	1.015 (0.677, 1.523)	
Location	Middle	1	0.271
	Middle-lower	1.219 (0.857, 1.735)	
Differentiation	Well	1	0.315
	Moderate	1.218 (0.644, 2.302)	0.544
	Poor	1.506 (0.813, 2.791)	0.193
Blood loss volume		1.001 (1.000, 1.002)	0.094
Age	≤60	1	0.046
	>60	1.401 (1.006, 1.950)	
Operation duration		1.004 (1.002, 1.006)	<0.001
Complications	No	1	0.037
	Yes	1.668 (1.031, 2.698)	
Neoadjuvant chemotherapy	No	1	<0.001
	Yes	1.938 (1.362, 2.756)	
Tumor size	≤5 cm	1	0.012
	>5 cm	1.581 (1.108, 2.257)	
Lymphovascular invasion	No	1	0.043
	Yes	1.476 (1.012, 2.151)	
T stage	T1	1	0.069
	T2	0.865 (0.183, 4.094)	0.855
	T3	2.093 (0.487, 8.995)	0.321
	T4	2.712 (0.790, 9.311)	0.113
N stage	N0	1	<0.001
	N1	2.234 (1.165, 4.284)	0.016
	N2	2.236 (1.149, 4.349)	0.018
	N3	4.416 (2.417, 8.067)	<0.001

## Discussion

Surgery is the only potentially curative method for patients with gastric cancer. The ideal surgical resection not only achieves the curative intent but also decreases postoperative morbidity and mortality. The long-term prognosis and postoperative quality of life should both be of great concern [12, 16, 17]. Considering that distal subtotal gastrectomy is associated with a better quality of life and lower morbidity and mortality, many surgeons recommend distal subtotal gastrectomy as the optimal procedure for lower-third gastric cancer based on previous reports [18–20]. However, at the moment, there is no consensus regarding the best extent of gastrectomy for middle-third gastric cancer. The only prospective randomized trial, performed in Italy, compared surgical morbidity and long-term prognosis between distal subtotal gastrectomy and total gastrectomy for patients with gastric cancer. However, only approximately 20% of patients in that study had middle-third gastric cancer [10, 21]. The conclusions in that study might not be appropriate for patients with middle-third gastric cancer. Until now, many surgeons have preferred to perform total gastrectomy for relatively later-stage middle-third gastric cancer to ensure the curativeness of the surgery. In our study, the proportion of relatively later-stage cases is higher in the total gastrectomy group than in the distal subtotal gastrectomy group. This result is concordant with previous reports [13, 22].

In our study, the total number of harvested lymph nodes is higher in the total gastrectomy group than in the distal subtotal gastrectomy group (*P* < 0.001). The primary reason is that we perform standard D2 lymph node dissection for locally advanced gastric cancer. During distal subtotal gastrectomy, the D2 lymph node dissection includes group 1, 3, 4sb, 4d, 5, 6, 7, 8a, 9, 11p and 12a lymph nodes. With regard to the total gastrectomy procedure, the scope of D2 lymph node dissection should include the above-mentioned groups and add group 2, 4sa, 10 and 11d lymph nodes [8, 15, 23]. Therefore, more harvested lymph nodes in the total gastrectomy group is reasonable. Although there are more harvested lymph nodes in the total gastrectomy group than in the distal subtotal gastrectomy group, the total gastrectomy procedure is more complicated than distal subtotal gastrectomy. Therefore, total gastrectomy consumes more time and induces more postoperative complications and longer postoperative hospital stay. In our study, the median operative durations in the total gastrectomy and distal subtotal gastrectomy groups are 240 min and 180 min, respectively (*P* < 0.001). Similarly, postoperative hospital stay is longer in the total gastrectomy group than in the distal subtotal gastrectomy group (*P* = 0.001). Notably, the complication rate is higher in the total gastrectomy group than in the distal subtotal gastrectomy group. Further analysis by splitting the different complications elucidates that the rate of anastomosis leakage is higher in the total gastrectomy group than in the distal subtotal gastrectomy group. Previous studies obtained similar conclusions [21, 24]. The main explanation for this is that the oesophago-jejunal anastomosis is more fragile than the gastro-jejunal anastomosis. The fragility of the oesophago-jejunal anastomosis leads to a higher rate of anastomosis leakage.

The effect on long-term survival of the different gastrectomy procedures has received the most attention for a long time. In our study, overall survival in the distal subtotal gastrectomy group is better than that in the total gastrectomy group, and the 5-year survival rates in the distal subtotal gastrectomy and total gastrectomy groups are 65% and 47%, respectively (*P* < 0.001). Given the imbalance in tumor staging between the groups, we perform stage-stratified subgroup analysis to compare the survival outcomes. In the stage-stratified analysis, the 5-year survival rates are comparable between the groups. The results demonstrate that the difference in 5-year survival rate between the distal subtotal gastrectomy and total gastrectomy groups is mainly induced by the imbalance of tumor staging. This result is also concordant with previous reports [22, 25].

To determine the prognostic factors for survival, univariate and multivariate analyses are performed. The results show that N stage (*P* < 0.001), tumor size (*P* = 0.012), presence of lymphovascular invasion (*P* = 0.043), operation duration (*P* < 0.001), complications (*P* = 0.037), age (*P* = 0.046) and usage of neoadjuvant chemotherapy (*P* < 0.001) are independent prognostic factors for survival. N stage, tumor size, presence of lymphovascular invasion and usage of neoadjuvant chemotherapy all directly or indirectly represent the degree of tumor progression and TNM staging. Thus, the result that these factors could affect survival outcomes is reasonable. In our study, complication is an independent prognostic factor for survival, as also reported in previous reports [10, 25]. Interestingly, the proximal resection margin and extent of gastrectomy are not prognostic factors for survival. In fact, many surgeons prefer to perform total gastrectomy for patients with middle-third gastric cancer to make sure that they achieve a safe proximal resection margin. In previous studies as well as in our study, only if an R0 resection is achieved would the distance from the resection margin to the tumor border not affect survival outcome [22, 26]. Meanwhile, the extent of gastrectomy is also not an independent factor for survival. In other words, distal subtotal gastrectomy would be a rational choice for patients with middle-third gastric cancer if an R0 resection could be achieved.

Some limitations exist in our study. This study is a retrospective study, and selection bias is difficult to avoid. For example, the proportions of patients with larger tumor size, lymphovascular invasion, neoadjuvant and adjuvant chemotherapy or later-stage disease are higher in the total gastrectomy group than in the distal subtotal gastrectomy group. These factors all represent a likely later-stage disease. The choice of the gastrectomy procedure’s extent is decided by surgeons, who usually choose total gastrectomy for patients with later-stage disease, as total gastrectomy seems to be a more effective and safe means to achieve a curative resection. However, stage-stratified subgroup analysis and multivariate analysis are performed in our study. The results of this study remain convincing. In the future, a prospective randomised controlled study is needed to further clarify the best extent of gastrectomy for patients with middle-third gastric cancer.

## Conclusions

For patients with middle-third gastric cancer, distal subtotal gastrectomy shortens operation duration and postoperative hospital stay and reduces postoperative complications. At the same time, the long-term survival of patients with distal subtotal gastrectomy is similar to that of those with total gastrectomy at the same stage. The extent of gastrectomy for middle-third gastric cancer is not an independent prognostic factor for survival.

## Abbreviations

UICC: Union for International Cancer Control
AJCC: American Joint Committee on Cancer
BMI: Body mass index
